# Conceptualization of the dynamics and synergistic partnership between the agri-food production chain and public health veterinarians

**Published:** 2015-03-15

**Authors:** Andrés Cartín-Rojas

**Affiliations:** *Department of Animal Production, Faculty of Agronomy, School of Natural and Exact Sciences, State Distance University, San José, Costa Rica.*

The agricultural value chain concept refers to the interaction between multiple manufacturing stages involved in the production process of a specific nourishment. The earned value of the final product is generated due to the operational or transactional costs through agro-food linear diagram and there is a livestock commodities dynamics pattern to meet expectations of consumers.^1^ This productive scheme takes and enforces many of the principles of quality management and incorporates them into the livestock production system. Accordingly, this approach aimed to guarantee the suitability of the final product is classified in primary chains involving the initial stages of production from breeding, nursing, weaning and growth of animals in livestock farms to the slaughter process. Secondary chains include all the subsequent stages of the primary chain, where the animal by-product is transformed, pitted, chopped, processed, stored, transported and retailed to the final consumer.^2^

The agreement on sanitary and phytosanitary measures (SPS agreement) of the world trade organization (WTO) recognizes the World Organization for Animal Health (OIE) standards as the reference codes for control and prevention of trans-boundary animal diseases and zoonoses, including food-borne zoonoses.^3^ Under the approach ''*from farm to fork*'' the OIE promotes harmonization of sanitary regulations for trade of animals and their products as well as the management of sanitary risks where veterinary professionals play a pivotal role. Thus, throughout the productive and value chain puzzle, veterinarians play key role in: 1) the application of risk analysis procedures,^4^ 2) quickly and efficiently detecting diseases listed by the OIE, including those that lead to a significant risk in terms of food safety and public health; and 3) international trade of livestock inputs, as veterinary certification is a crucial requirement to export animal products. Thereby, there is a mutualistic and synergistic partnership between the agri-food production chain structure and public health veterinarian, where the functionality and competitiveness of the agricultural value chain structure to access the global markets, is a primary responsibility of veterinarians. Whereas, the interaction and partnership of each stake-holder across the horizontal chain structure ensures the integration, strengthened and correct implementation of public health policies at public and private level carried out by the veterinary services. For example, by evaluating the microbiological risks and estimating the appropriate level of protection (ALOP), food safety objectives (FSO) and performance objectives (PO); or enforcing and developing traceability programs for livestock or animal by-products.^5^

Moreover, agro-terrorism and bioterrorism prevention, changes in socio-demographic trends in developing countries, dietary and agricultural exporter patterns promoted by globalization of livestock markets, will evolve with a consistent and progressive parallelism towards more robust and strong food security surveillance schemes.^6^ Likewise, one of the most significant impacts of globalization is the need to upgrade veterinary services to promote and protect human and animal health and adjust local nourishment production to international trade models under the SPS Agreement standards. Therefore, agricultural value chains constitute an alternative to enhance the utilization of resources, afford compliance with environmental protocols, improve sanitary management of trans-boundary animal diseases and food-borne zoonoses, increase access to global markets, strengthen rural development paths, and establish an integral framework for the exchange of tools to reduce poverty, food insecurity and social inequality.

In this scenario, veterinary professionals serve as catalysts to bolster rural economies by enabling the development of a sustainable livestock activity and guarantee food hygiene.^7^ Hence, it is essential to strengthen both academic and professional training programs at undergraduate and postgraduate level of veterinary public health and food safety programs,^8^ where particular importance should be granted to manufacturing and value chain structures, as holistic and trans-disciplinary systems that contribute in a practical and tangible way to safeguard and improve the competitiveness of our local agri-food markets.
